# 

**Published:** 1978

**Authors:** 


Contents

3 Obituary—
Robert V. Cooke, M.D., Ch.M., F.R.C.S.

5 Richard Bright's Reports of Medical Cases (1827): A sesquicentennial note
Jeffrey Boss

7 Medical Care—more or less?
H. G. Mather, M.A., M.D., F.R.C.P.

